# Upstream of N-Ras (Unr/CSDE1) Interacts with NCp7 and Gag, Modulating HIV-1 IRES-Mediated Translation Initiation

**DOI:** 10.3390/v14081798

**Published:** 2022-08-17

**Authors:** Nedal Taha, Sarwat Zgheib, Kamal Kant Sharma, Nicolas Humbert, Emmanuel Boutant, Pascal Didier, Yves Mély, Eleonore Real

**Affiliations:** 1 UMR 7021 CNRS, Laboratoire de Bioimagerie et Pathologies, Université de Strasbourg, Faculté de Pharmacie, 67401 Illkirch-Graffenstaden, France; 2 American Red Cross-HLA Laboratory, 180 Rustcraft Road, Suite 115, Dedham, MA 02026, USA; 3 Centre for Bioimaging Sciences, Department of Biological Sciences, National University of Singapore, 14 Science Drive 4, Singapore 117557, Singapore

**Keywords:** HIV, nucleocapsid, NCp7, Unr, Gag, IRES

## Abstract

The Human Immunodeficiency Virus-1 (HIV-1) nucleocapsid protein (NC) as a mature protein or as a domain of the Gag precursor plays important roles in the early and late phases of the infection. To better understand its roles, we searched for new cellular partners and identified the RNA-binding protein Unr/CSDE1, Upstream of N-ras, whose interaction with Gag and NCp7 was confirmed by co-immunoprecipitation and FRET-FLIM. Unr interaction with Gag was found to be RNA-dependent and mediated by its NC domain. Using a dual luciferase assay, Unr was shown to act as an ITAF (IRES trans-acting factor), increasing the HIV-1 IRES-dependent translation. Point mutations of the HIV-1 IRES in a consensus Unr binding motif were found to alter both the IRES activity and its activation by Unr, suggesting a strong dependence of the IRES on Unr. Interestingly, Unr stimulatory effect is counteracted by NCp7, while Gag increases the Unr-promoted IRES activity, suggesting a differential Unr effect on the early and late phases of viral infection. Finally, knockdown of Unr in HeLa cells leads to a decrease in infection by a non-replicative lentivector, proving its functional implication in the early phase of viral infection.

## 1. Introduction

The human immunodeficiency virus type-1 (HIV-1) belongs to the *Lentivirus* genus of the *Retroviridae* family. Its positive-stranded RNA genome (gRNA) resulting from the polymerase II-mediated transcription of the integrated viral genome (vDNA) is capped and polyadenylated. This gRNA is thus used both for packaging into new virions and translation of the viral precursor proteins. Interestingly, the highly structured 5′ non-coding region (5′-UTR) of HIV-1 [1,2,3] has first been shown to block ribosomal scanning in vitro [4], but it was later shown that it is not the case in cultured cells, thus showing that the cap-dependent translation is dependent on cellular factors and physiological conditions [5,6]. However, the cap-dependent translation is not the sole mechanism described for HIV-1 translation. Internal Ribosome Entry Site (IRES)-dependent translation has been described for HIV-1, especially under conditions where the cap-dependent translation is impaired. In fact, the highly structured 5′ extremity of the HIV-1 gRNA harbors two IRESs [7]. IRESs were first described among picornaviruses before their identification in other viral and cellular Ribonucleic acids (RNAs). IRESs are highly structured RNA domains found most often in the 5’ non-coding region of messenger RNAs which allow the 40S ribosomal subunit recruitment without the need for the 5′-cap structure or cap-dependent translation mandatory initiation factors. For HIV-1, the first functional IRES described is localized within the gag Open Reading Frame (ORF) [8]. It drives the expression of both the full length and a shorter isoform of the Gag polyprotein (Gagp40) from an alternative AUG start codon. This IRES unconventionally drives translation from an AUG localized at its 5′ extremity and is also found to be functional in other retroviruses, including HIV-2 [9,10,11]. A second, more conventional IRES is found in the 5′-Untranslated Region (UTR) of the viral gRNA and thus in all HIV-1 messenger RNA (mRNA) more precisely between nucleotides 1–336, with nucleotides 104–336 representing the minimal functional sequence [12]. This genome region includes several secondary structures implicated in genome transcription, encapsidation, dimerization and reverse transcription, including Trans Activation Response (TAR), poly(A), Primer Binding Site (PBS), Dimerization Initiation Sequence (DIS) and the packaging signal Psi [12]. Since this IRES is not functional in HIV-2, its functionality in HIV-1 has been a subject of a long debate since its first description in 1996 by Brasey et al. [12]. The contradictory results observed for this IRES are in part explained by the fact that it is not functional in a rabbit reticulocyte lysate (RRL) in vitro system [4]. However, it becomes functional upon RRL supplementation by cellular extracts, especially from HeLa cells blocked in G2/M [13,14]. Additionally, it also becomes functional when used in vitro with HeLa cells based translation extracts [12], in cell-based assays [12,15,16,17,18] or in *Xenopus laevis* oocytes [13,14,18]. This underlines its dependence on cellular factors, as it was already shown for Polio- or Rhinoviruses IRESs [19,20] or for the cellular IRES from the Apoptotic protease activating factor 1, Apaf1 mRNA [21].

HIV-1 IRES requirement for IRES trans-acting factors (ITAFs) is supported by the literature as cap- and IRES-dependent mechanisms of translation initiation were shown to be active at different levels according to the cellular state and the progress of the infection [22,23]. The IRES is particularly active when the cell is blocked in G2/M as is the case during HIV-1 infection [15,22,23,24].

So far, several ITAFs have been identified for viral or cellular IRESs. Although their modes of action are poorly known, they are suggested to act as RNA chaperones rearranging the secondary structures of the IRES or as adaptor proteins bridging the RNA to the proteins of the 40S ribosome subunit [25]. In any case, their ability to regulate the activity of IRESs and their variable level of expression in different cell types is one of the leading explanations for the difference in activity observed for a given IRES across various cell types [26,27,28]. One of these ITAFs, the cytoplasmic protein Unr (Upstream of N-Ras), contains 5 Cold Shock Domains (CSDs) which bind nucleic acids (NAs) as well as proteins [29,30]. Unr, also called CSDE1, is known to act as a cytoplasmic RNA binding protein to regulate its target mRNA stability and translation [31,32]. The IRES regulating action of Unr has been demonstrated for the Polio- and Rhinoviruses IRESs, in addition to cellular IRESs, including Apaf1 and its own IRES [33,34,35,36]. As an ITAF of the HIV-1 IRES and being overexpressed in cells blocked in G2/M, Unr could explain the sustained synthesis of HIV-1 proteins during the course of an HIV-1 infection despite the cell block in G2/M or the cleavage by HIV-1 protease of the canonical cap-dependent initiation factor eIF4G.

The HIV-1 nucleocapsid protein is present in two forms in the virus particle and the infected cell, as a domain of the polyprotein Gag (NC-Gag) and as a product of cleavage of Gag (mature form, NCp7). Because of its ability to bind to Nas [37,38,39], NC/NCp7 is a major player in both the early and late stages of the viral cycle [40,41,42]. In the early phase, it protects the incoming gRNA from cellular nucleases, chaperones Nas, during reverse transcription and probably promotes the integration of the provirus [43]. In the late phase, it participates in the selective encapsidation and dimerization of the gRNA into the viral particles [44,45,46,47,48]. The encapsidation results in the formation of high-order Gag multimers on the gRNA and the concomitant trafficking of this ribonucleoprotein to the cell plasma membrane [44,49,50,51]. The central role of NC-Gag/NCp7 in infection is emphasized by the drastic loss of infectivity observed after single-point mutations in either of its two zinc fingers [52,53].

Interestingly, Unr has been found as a partner of the NCp7 protein in affinity purification analysis coupled to mass spectroscopy [54]. In the present work, we found by biochemical and microscopy approaches that Unr acts as an ITAF for the HIV-1 IRES and that this activity is regulated by NC-Gag, but not by NCp7. We further showed that Unr interacts with NCp7 and NC-Gag. Finally, a knockdown of Unr by small interfering RNA (siRNA) was found to decrease HeLa cells infection by pseudotyped HIV-1 pseudoparticles.

## 2. Materials and Methods

### 2.1. Mammalian Cell Culture

HeLa cells and 293T cells were maintained in Dulbecco’s modified Eagle medium (ThermoFisher Scientific, ref 21885025 and 31966047 respectively, Waltham, MA, USA) supplemented with 10% fetal calf serum (Invitrogen Corporation, Villebon sur Yvette, France) and a mixture of penicillin (100 U/mL) and streptomycin (10 U/mL) antibiotics (Lonza DE17-602E) at 37 °C in a 5% CO_2_.

### 2.2. Plasmids and Proteins

The dlHIV-1, dlVAR2 and dl-Apaf (pRAF) were generous gifts from M. Vallejos and A. Willis and were described earlier [18,21]. The plasmid constructs for Vesicular Stomatitis Virus G protein (VSV-G) pseudotyped HIV-1 particles, namely, pMD2.G and pCMV-dR8.91, were obtained from Addgene. pSicoR-luciferase was obtained by the replacement of the eGFP cDNA in pSicoR-eGFP by the firefly luciferase cDNA between the NheI and EcoRI cloning sites. In PEF-Flag-Unr (a generous gift from J. Sablon), the Unr gene is cloned with its N-terminus fused to a Flag sequence and expressed under the control of an EF-1a promoter. peGFP-Unr and pmCherry-Unr were cloned by Gateway^®^ cloning from PEF-Flag-Unr, using the following primers (AttB1-Unr: 5′-GGGGACCACTTTGTACAAGAAAGCTGGGTTTAGTCAATGACACCAGCTTGAC-3′ and AttB2-Unr-Stop: 5′-GGGGACCACTTTGTACAAGAAAGCTGGGTTTAGTCAATGACACCAGCTTGAC-3′) into the destination vector peGFP-C1-GW. IRES mutants were generated by site-directed mutagenesis using the Phusion site-directed mutagenesis kit (ThermoFisher Scientific, F541, Waltham, MA, USA) according to the manufacturer’s instructions with the primers listed in Table 1 purchased from Sigma. Gag, GagΔNC and NCp7 were cloned into a pcDNA3.1 vector [55].

The biotinylated labeled NCp7 (Biotin-KQRGNFRNQRKNVKCFNCGKEGHTARNCRAPRKKGCWKCGKEGHQMKDCTERQAN) was synthesized by solid-phase peptide synthesis on a 433A synthesizer (ABI, Forster City, CA, USA) as described previously [56]. Peptides were stored lyophilized. The zinc-bound form of the peptides was prepared by dissolving them in water, adding a 2.5-fold molar excess of zinc sulfate, and raising the pH to its final value by adding buffer. The pH was increased to its final value only after the addition of zinc to avoid the oxidation of the zinc-free peptide. The peptide concentration was determined by using an extinction coefficient of 5.7 × 10^3^ M^−1^ cm^−1^ at 280 nm.

### 2.3. Dual Luciferase Assays

HeLa cells were seeded in 12 well plates with a density of 8 × 10^4^ cells/ well. Cells were transfected 24 h post seeding, using jetPEI™ Polyplus Transfection™ according to the manufacturer’s protocol, with 200 ng of the dual luciferase IRES construct and 250 ng of each construct coding for Gag, GagΔNC, NCp7 cloned in pcDNA3.1 and/or Unr-Flag cloned in pEF vector. Total plasmid DNA was completed by an empty vector to 700 ng. Firefly and *Renilla* luciferase activities were measured with the Dual-Luciferase^®^ Reporter Assay System (Promega, E1910, Madison, WI, USA) according to the manufacturer’s protocol, using a luminometer (Berthold technologies, TriStar LB941, Bald Wildbad, Germany), 24 h post transfection. Briefly, cells were lysed in 1× lysis buffer (Promega) (250 μL/well) for 15 min at room temperature, and 20 μL of the sample was introduced in each well of a 96-well, flat-bottomed, white plate (Greiner Bio-One, Kremsmünster, Austria). Then, 70 μL of luciferase assay reagent II (LARII) was injected into each well, and the luminescent signal was accumulated for 10 s. Then, the firefly luciferase reaction signal was quenched by adding 70 μL Stop & Glo^®^ Reagent to each well, and the *Renilla* luciferase activity signal was measured for 10 s. Statistics were done using the student *t*-test.

### 2.4. Co-Immunoprecipitation

293T cells were seeded in a 6-well plate at a concentration of 60 × 10^4^ cells/well 24 h before cotransfection with 2 µg of each plasmid coding for the proteins of interest, and 48 h post-transfection, cells were washed in 1× PBS and lysed in RIPA buffer (Tris-HCl 10 mM pH = 7.5, NaCl 150 mM, EDTA 1 mM, and 1% NP40, complete™ Mini-EDTA free protease inhibitor Cocktail Tablets (Roche, 11836170001, Basel, Switzerland)). After centrifugation to remove cell debris, the protein concentration was assessed by a Bradford assay. An input fraction (30 μg) was kept to check the protein expression level, and the equivalent of 1 mg of lysate was incubated 2 h at 4 °C with 1 μg of anti-Flag^®^ antibody (Merck, F1804, Rahway, NJ, USA) on a rotating wheel. In the case of Rnase A treatment, the lysate was incubated for 30 min at room temperature with 100 μg/mL of RNAse A (Sigma-Merck, R6513, Burlington, MA, USA) before the addition of the antibody. After equilibration, 50 µL of Dynabeads protein A (ThermoFisher Scientific, 10002D) were added, and the mixture was incubated for 1 h at 4 °C. Beads were washed 3 times with cold 1× lysis buffer, resuspended in Laemmli sample buffer (Bio-Rad, 161-0747, Hercules, CA, USA), boiled for 5 min, and analyzed by Western blot.

### 2.5. Western Blots

Protein samples were electrophoresed on a 12% SDS-PAGE gel. Subsequently, proteins were transferred onto a polyvinylidene difluoride membrane (Amersham, RPN303F), and blots were probed with mouse monoclonal antibody anti-Cap24 Gag (AIDS Reagent Program, 6521 #24-4; [57,58], anti-eGFP (Proteintech, 66002-1, Rosemont, IL, USA), anti-Unr (Proteintech, 13319-1-AP), or anti-GAPDH (Merck, MAB374). After three PBS washes, secondary anti-mouse or anti-rabbit antibodies (Promega WB401B and W402B) conjugated to the horseradish peroxidase were added to the membrane, and proteins were visualized by a homemade chemioluminescent ECL system on an Image Quant LAS 4000 apparatus (GE healthcare, Chicago, IL, USA).

### 2.6. Confocal Microscopy

HeLa cells were seeded on a glass coverslip in 12 well plates with a density of 8 × 10^4^ cells/ well, 24 h before being transfected with the appropriate plasmids as described above. Cells were washed with PBS and fixed with 4% Paraformaldehyde (PFA) in 1× PBS for 15 min at room temperature 24 h post transfection. After fixation, cells were washed three times with 1× PBS and mounted on slides using Prolong Gold Antifade Reagent (ThermoFisher Scientific, 36930). The cellular localization of the proteins of interest coupled to eGFP or mCherry was visualized by confocal microscopy with a Leica SPE equipped with a 63 × 1.4 NA oil immersion objective (HXC PL APO 63×/1.40 OIL CS). The eGFP images were obtained by scanning the cells with a 488 nm laser line and using a 500- to 555-nm band-pass for emission. For the mCherry images, a 561 nm laser line was used with a 570–625 nm band-pass. Images were analyzed by the Image J software (version 1.8.0, NIH, Bethesda, MD, USA).

### 2.7. Fluorescence Lifetime Imaging Microscopy (FLIM)

FLIM measurements were performed using the time-correlated single photon counting approach on a homemade two-photon excitation scanning microscope based on an Olympus IX70 inverted microscope with an Olympus 60 × 1.2 NA water immersion objective operating in the descanned fluorescence collection mode [59,60]. Two-photon excitation at 900 nm was provided by a mode-locked titanium-sapphire laser (Spectra-physics, Milpitas, CA, USA) or an Insight DeepSee (Spectra Physics) laser. Photons were collected using a set of two filters: a short-pass filter with a cutoff wavelength of 680 nm (F75-680; AHF, Tübingen, Germany) and a band-pass filter of 520 ± 17 nm (F37-520; AHF, Tübingen, Germany). The fluorescence was directed to a fiber-coupled APD (SPCM-AQR-14-FC; PerkinElmer, Waltham, MA, USA), which was connected to a time-correlated single photon counting module (SPC830, Becker & Hickl, Berlin, Germany). Typically, the samples were continuously scanned for about 60 s to achieve the appropriate photon statistics in order to reliably analyze the fluorescence decays. Moreover, to reach the Nyquist–Shannon sampling criteria, we carried out FLIM measurements using a 20 μm × 20 μm scale and 256 pixels × 256 pixels. Data were analyzed using a commercial software package (SPCImage v2.8; Becker & Hickl, Berlin, Germany). For Förster resonance energy transfer (FRET) experiments, the FRET efficiency © was calculated according to E = −1 − (τ_DA_/τ_D_), where τ_DA_ is the lifetime of the donor (eGFP) in the presence of the acceptor (mCherry) and τ_D_ is the lifetime of the donor in the absence of the acceptor.

### 2.8. Viral Production and Quantification

Stocks of VSV-G pseudotyped HIV-1 pseudoparticles were prepared by transfecting 3 µg of pMD2.G, 12 µg of pSiCoR-luciferase, and 6 µg of pCMV-dR8.91 vectors into 293T cells using the standard JetPEI transfection protocol (PolyPlus, Illkirch-Graffenstaden, France). At 48 h post transfection, cell culture supernatants were collected, clarified on a 0.45 µm PVDF filter (Millipore SLHV033RS, Burlington, MA, USA), and concentrated twice by a Vivaspin20 centrifugal concentrator (Sartorius, VS2031, Göttingen, Germany). Supernatants containing pseudoparticles were stored in 500 µL aliquots at −80 °C. In parallel, viral stocks were titrated by anti-p24 enzyme-linked immunosorbent assay (ELISA) according to the manufacturer’s instructions (Innotest^®^ HIV Antigen mAb, Fujirebio, Tokyo, Japan).

### 2.9. Viral Infection

HeLa cells were seeded in 6-well plates at a concentration of 5 × 10^4^ cells/well. The next day, cells were either transfected with 50 nM of control siRNA or Unr siRNA in order to knock down Unr (LU-015834-00-0002 and D-001810-10-20, Dharmacon, Lafayette, CO, USA), using the Jet Prime transfection protocol (PolyPlus transfection, Illkirch-Graffenstaden, France). Then, 48 h post transfection, cells were infected with the viral pseudoparticle supernatant equivalent to 360 ng of p24/well, and 1 µM of AZT (zidovudine) was used as a positive control, whereas non-infected cells were used as negative control. After 8 h, the medium was changed and replaced by a virus-free medium, and the cells were kept for 48 h after infection, before lysis and RNA extraction.

### 2.10. Quantitative PCR (qPCR)

RNA was extracted with the RNeasy Plus Mini kit (Qiagen, Hilden, Germany) according to the manufacturer’s instructions and was transcribed into cDNA using the iScript ^TM^ Select cDNA Synthesis Kit (Bio-Rad). qPCR master mixes were prepared in a MicroAmp^®^ Optical 96-well Reaction Plate (Applied Biosystems, Waltham, MA, USA) with 2× Fast SYBR^®^ Green Master Mix (Applied Biosystems, 4385612) according to the manufacturer’s recommendations. Plates were sealed with an Optical Adhesive Cover. qPCR reactions contained 300 nM forward and reverse primers and 50 ng/μL reverse transcribed cDNA template in a total reaction volume of 20 μL. Amplification of cDNA was carried out by real-time quantitative PCR and detected using an ABI 7000 Sequence Detection System (Applied Biosystems). Gene expression was normalized to housekeeping genes, and samples were run alongside RT-negative cDNA (produced without reverse transcriptase) and H_2_O controls.

Cycle thresholds (C_T_) were determined per transcript in triplicate with the following probes:

Luciferase Forward: 5′-TGAGTACTTCGAAATGTCCGTTC-3′

Luciferase Reverse: 5′GTATTCAGCCCATATCGTTTCAT-3′

18S Forward: 5′TGTGGTGTTGAGGAAAGCAG-3′

18S Reverse: 5′TCCAGACCATTGGCTAGGAC-3′

Relative levels of mRNA gene expression were calculated using the 2^−ΔΔCt^ method [61].

## 3. Results

### 3.1. HIV-1 IRES Activity Is Stimulated by Unr

To provide further information in the debate on HIV-1 IRES activity and functional relevance [5,6,12,14,17,18,22,23,62], we investigated the putative role of upstream of N-ras (Unr) as an ITAF of the HIV-1 IRES. We selected Unr because (i) it is an ITAF for polio- and rhinoviruses as well as for cellular IRESs whose mRNA is capped and polyadenylated similarly to the one of HIV-1, (ii) its expression is cell cycle regulated, showing overexpression in G2/M and (iii) it potentially interacts with both Gag and NCp7 [54].

**Figure 1:**
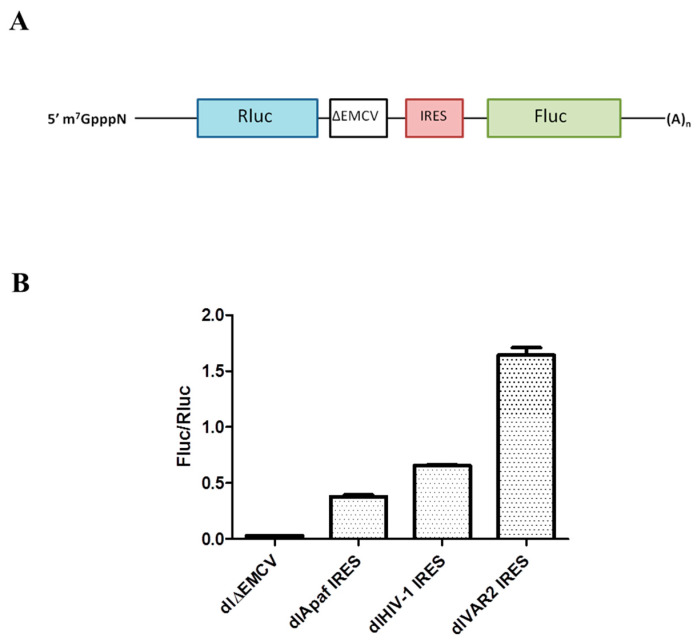
IRES activity measured by a dual luciferase assay. (**A**) Scheme of the construct used to perform the dual luciferase test. The IRES of interest is inserted into a dual luciferase construct (dl) between an upstream Renilla luciferase gene (Rluc) and the ΔEMCV defective IRES and a downstream firefly luciferase gene (Fluc). (**B**) Activity test in HeLa cells of dlApaf-1 IRES, dlHIV-1 IRES and dlVAR2 IRES. The dlΔEMCV IRES was used as a negative control. The IRES activity is monitored by the ratio of Fluc/Rluc luciferase activities. Measurements were performed in HeLa cells 24 h post transfection. Histograms represent the mean ± SEM for at least 3 independent experiments done in triplicate.

The HIV-1 IRES functionality was tested in a well-established dual luciferase system [18], similar to the one used by Brasey and Gendron [12,15]. In this system, the first 336 nucleotides of the pNL4.3 HIV-1 clone are inserted into a dual luciferase construct (dl) between an upstream *Renilla* luciferase gene (Rluc) and a downstream firefly luciferase gene (Fluc). A defective encephalomyocarditis virus IRES sequence (ΔEMCV) was introduced between the two cistrons in order to prevent translation of the downstream ORF from reinitiation or readthrough of ribosomes (Figure 1A). After transient transfection and CMV promoter-driven transcription in HeLa cells, the resulting bicistronic mRNA is translated via a cap-dependent mechanism for the Rluc gene and an IRES-dependent one for the Fluc gene. The ratio of the Fluc/Rluc activities reflects the IRES activity normalized to the cap-dependent one. These bicistronic mRNAs offer the advantage of allowing the IRES to be studied in the absence of virus infection, thus eliminating potential interferences from the viral replication cycle or from other regions of the gRNA which could influence the HIV-1 IRES [12,62,63].

**Figure 2:**
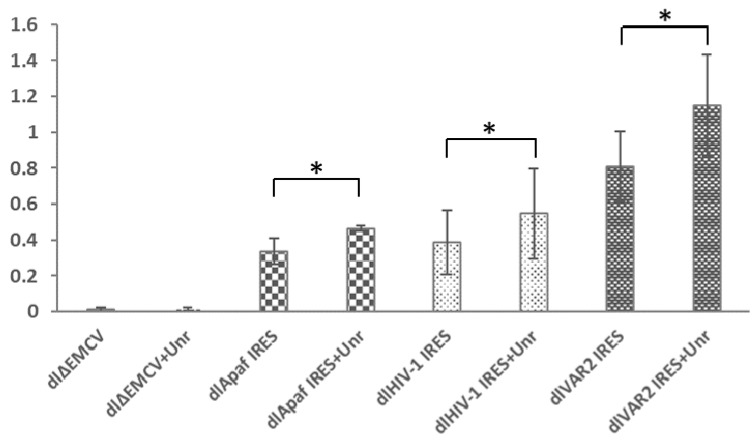
Effect of Unr overexpression on the activity of HIV-1 IRES. The IRES activity is monitored by the ratio of Fluc/Rluc luciferase activities in the dual luciferase assay. Two variants of HIV-1 IRES (HIV-1 and VAR2) are tested. The ΔEMCV and Apaf-1 IRESs are given as negative and positive controls, respectively. Measurements were performed in HeLa cells 24 h post transfection. Histograms represent the mean ± SEM of at least 3 independent experiments done in triplicate. * *p* < 0.05.

The activity of the HIV-1 IRES was assessed in this system in comparison to two other IRESs, namely the IRES of a clinical HIV-1 isolate (VAR2), previously described as being four times more active than the HIV-1 IRES in HeLa cells [18] and the cellular IRES of Apaf-1 mRNA which is also active in HeLa cells [21,64]. Using HeLa cells transiently transfected by the dl constructs, we found that all IRESs are active (Figure 1B), with the HIV-1 IRES being 1.7 times more active than the one of Apaf-1 but 2.5 times less active than the one of the clinical variant VAR2. We next investigated the effect of Unr overexpression on these different IRESs. Cells were thus co-transfected with the corresponding dl constructs and pEF-Flag-Unr, and luciferase activity was quantified 24 h post transfection. With the exception of dlΔEMCV, all IRESs presented an increase in their activity of around 40% (Figure 2). In the case of Apaf-1, this Unr-mediated increase was expected since Unr was shown to be a stimulating ITAF of the Apaf1 IRES [35]. Like Apaf-1 IRES, Unr increases the translation activity of HIV-1 IRES, suggesting that Unr acts as an ITAF of this IRES.

### 3.2. Identification of Unr Binding Site in the HIV-1 IRES

An in vitro selection approach (SELEX) has identified two purine-rich unstructured consensus sequences (Pu)_5_AAGUA(Pu) or (Pu)_8_AAC(Pu)_3_ as preferential binding sites for Unr [65]. With the aim of mapping the Unr binding site on the HIV-1 IRES, we searched for the presence of such sequences in regions predicted to be single-stranded within the minimal active pNL4.3 HIV-1 IRES sequence localized between nucleotides 104–336. We identified two sequences of this type, namely the (Pu)_2_(Py)(Pu)_2_AAGUA(Pu) sequence at the level of nucleotides 205–215 and the (Pu)_8_AACA(Pu)_3_ sequence at the level of nucleotides 183–197 [14].

Based on the SELEX analysis mentioned before, we designed two IRES mutants of nucleotides located between positions 210 and 214 in order to reduce the binding affinity to Unr. To do so, we selected for each position nucleotides that were never found in the RNAs selected as Unr partner in the SELEX selection, i.e., C or U at positions 210, 211 and 214, A or C at position 212 and finally, G or A at position 213 [65]. The mutations were inserted into the HIV-1 IRES cloned in the dual luciferase construct to give the mutU and mutC constructs (Table 2).

**Figure 3:**
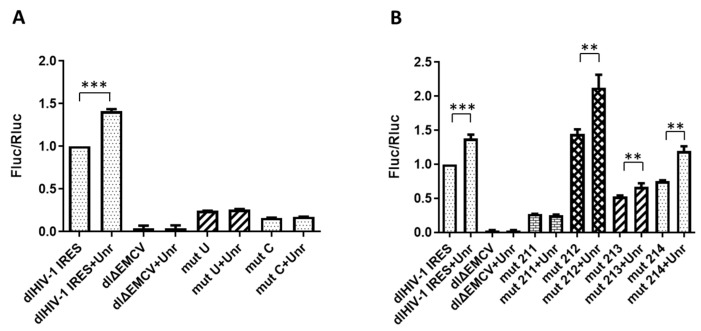
Determination of the activities of mutated HIV-1 IRES with/without Unr overexpression. (**A**) MutU and mutC IRES activities with/without Unr overexpression. (**B**) IRES activities of mut211, mut212, mut213 and mut214 with/without Unr overexpression. The IRES activity is expressed by the ratio of Fluc/Rluc luciferase activities in the dual luciferase assay. Measurements were performed in HeLa cells 24 h post transfection. The ratios are normalized to the ratio of the WT HIV-1 IRES without Unr overexpression. The construct dlΔEMCV corresponds to an inactive IRES control. Histograms represent the mean ± SEM of at least 3 independent experiments done in triplicate. ** *p* < 0.01; *** *p* < 0.001.

The two IRES mutants, mutU and mutC, tested in the dual luciferase assay showed a dramatic decrease in IRES activity of, respectively, 75% and 84% in respect with the wild-type (WT) level in the absence of Unr overexpression (Figure 3A). When Unr was overexpressed, no IRES activity increase was seen with both mutants, while a 40% increase was observed for the WT IRES. This loss of sensitivity for the two IRES mutants to Unr overexpression supported our hypothesis that the 210–214 sequence may be involved in Unr binding. To exclude the possibility that the observed effect may be due to the destabilization of the IRES structure by the simultaneous mutation of residues 210–214, we generated a set of single-point mutated IRESs referred to as mut211, mut212, mut213 and mut214 (Table 2). Such single-point mutations have been shown to not significantly destabilize the IRES structure [14,15,18,66].

The single-point mutants showed different levels of IRES activity and response to Unr. Mut211 exhibited a strong reduction (73%) in IRES activity and was not responsive to Unr overexpression (Figure 3B). A less important reduction in the IRES activity was observed for mut213 and mut214 with, respectively, 52% and 75% of the WT activity. They also respond to Unr overexpression, showing an increase in IRES activity of 27% and 60%, respectively. Interestingly, mut212 IRES activity was 44% stronger than the one of the WT IRES in the absence of Unr over-expression. Moreover, its response to Unr overexpression was comparable to the WT one (48% for mut212 vs. 40% for the WT; Figure 3B).

Taken together, our data strongly suggest that the 210–214 sequence plays an important role in the HIV-1 IRES activity, being an Unr binding site and that Unr can modulate HIV-1 IRES activity by binding at this site.

### 3.3. Opposite Effects of Gag and NCp7 on Unr-Promoted HIV-1 IRES Activity

**Figure 4:**
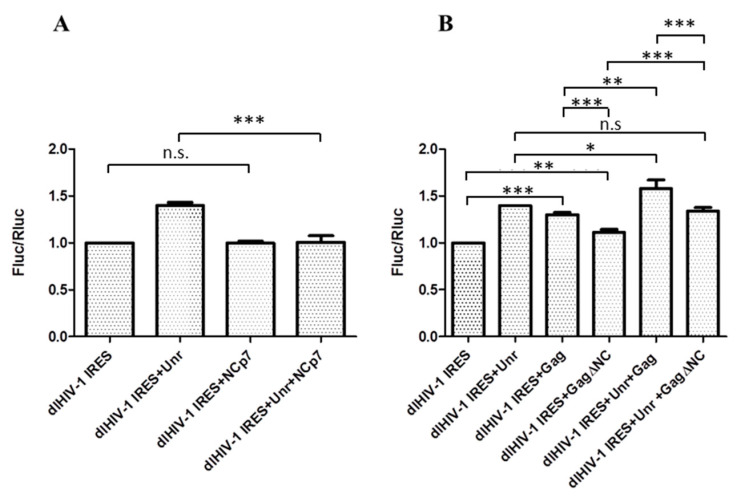
Effect of NCp7 (**A**) and Gag or GagΔNC (**B**) on HIV-1 IRES activity with or without Unr overexpression. The IRES activity is expressed by the ratio of luciferase activities Fluc/Rluc in the dual luciferase assay. The measurements were performed in HeLa cells 24 h post transfection. Histograms represent the mean ± SEM of at least 3 independent experiments done in triplicate. n.s.—non-significant. * *p* < 0.05; ** *p* < 0.01; *** *p* < 0.001.

As the HIV-1 5′-UTR harbors several functional domains, including the primary gRNA packaging signal, psi [67], and the primer binding site (PBS) [68], which are targets of the NC domain of Gag and NCp7, we wondered whether HIV-1 IRES activity can be modulated by NCp7 or Gag. Therefore, we investigated the effect of NCp7 and Gag overexpression on HIV-1 IRES activity using our dual luciferase system. While NCp7 overexpression did not affect HIV-1 IRES activity, it counteracted the stimulating effect of Unr overexpression on this activity (Figure 4A). In contrast to NCp7, Gag overexpression increased the IRES activity by about 30%, close to the level of Unr stimulation. When both Gag and Unr were overexpressed, their effects on IRES activity were clearly additive, leading to a 60% increase with respect to the control without any overexpression (Figure 4B). As the main contributor to the nucleic-acid-binding activity of Gag is the NC domain, which is endowed with a potent nucleic acid chaperone activity [69,70,71,72], it could be hypothesized that the stimulatory effect of Gag on the IRES activity is driven by its NC domain. We thus overexpressed a Gag mutant where the NC domain was deleted (GagΔNC) with the dual luciferase construct. In line with our hypothesis, this mutant only increased by 11% the HIV-1 IRES activity. Overexpression of both Unr and GagΔNC gave the same HIV-1 IRES activity as Unr alone, in line with the need of the NC domain for the Gag stimulation effect.

### 3.4. NCp7/Gag and Unr Co-Localize in the Cytoplasm

**Figure 5:**
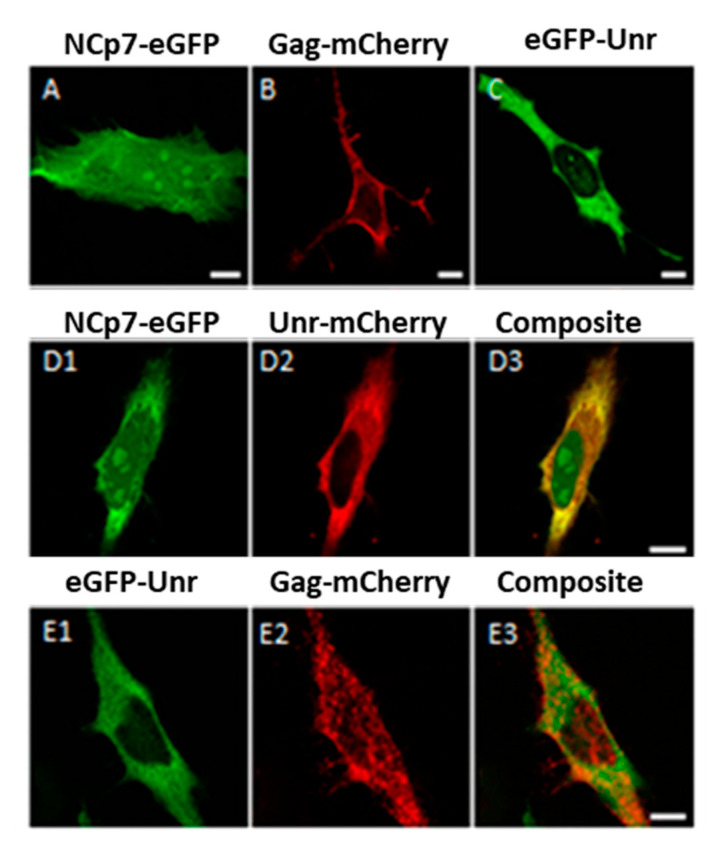
Colocalization of NCp7 and Gag with Unr in HeLa cells. (**A**–**C**) HeLa cells were transfected with a single plasmid coding for NCp7-eGFP (**A**), Gag-mCherry (**B**), eGFP-Unr (**C**) 24 h before being fixed with paraformaldehyde and imaged by confocal microscopy. (**D**,**E**) HeLa cells were doubly transfected with constructs overexpressing NCp7-eGFP and Unr-mCherry (**D1**,**D2**) or eGFP-Unr and Gag-mCherry (**E1**,**E2**). (**D3**,**E3**) show the composite images of (**D1**,**D2**) and (**E1**,**E2**), respectively. The bar scale represents 10 µm.

Since we demonstrated that Gag and NCp7 can modulate the Unr effect on HIV-1 IRES, and since Jäger et al. identified, by mass spectrometry, Unr as being part of affinity-purified protein complexes bound to NCp7 and Gag overexpressed in HEK cells [54], our next objective was to confirm the interaction between Unr and NCp7 and/or Gag. In order to observe the possible colocalization of NCp7/Gag and Unr, we transiently expressed Unr-mCherry with NCp7-eGFP or eGFP-Unr with Gag-mCherry in HeLa cells. The cellular distributions of the fusion proteins were in accordance with the known distributions of the three proteins, with NCp7 being found in all cell compartments with a preferential localization in the cytoplasm and the nucleoli (Figure 5A), Gag accumulating at the cell plasma membrane (Figure 5B) and Unr being mainly cytoplasmic (Figure 5C). Interestingly, Unr and NCp7 were both observed in the cytoplasm in which they colocalized (Figure 5(D3)). In contrast, no significant colocalization was observed for Gag, which is rapidly transported to the membrane after its translation (Figure 5(E3)). Neither Gag nor NCp7 expression changed Unr localization (compare Figure 5 panels D2 and E1 with panel C).

### 3.5. NCp7 Interaction with Unr Visualized by FRET-FLIM

**Figure 6:**
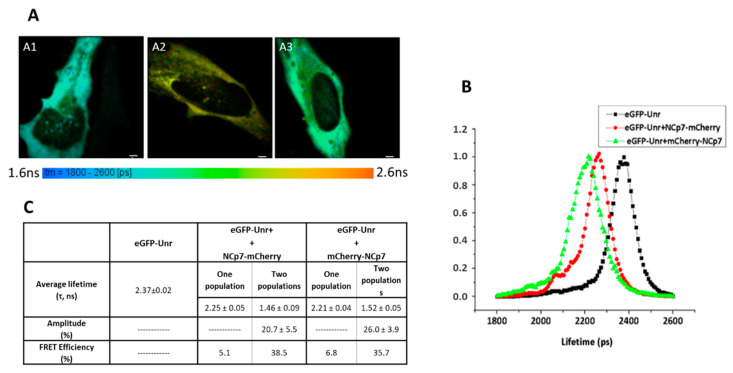
Unr/NCp7 interaction monitored by FRET-FLIM. HeLa cells were transfected with DNA constructs encoding eGFP-Unr (**A1**) and NCp7-mCherry (**A2**) or mCherry-NCp7 (**A3**). Live cells were imaged 24 h post-transfection. The eGFP fluorescence lifetime was determined using a single-exponential model and converted into a color code ranging from blue (1.6 ns) to red (2.4 ns). (**B**) Distributions of τ values expressed in ps for cells expressing eGFP-Unr alone (black); eGFP-Unr with NCp7-mCherry (red) or eGFP-Unr with mCherry-NCp7 (green). (**C**) Fluorescence lifetimes of eGFP-Unr, in the presence of NCp7-mCherry or mCherry-NCp7, analyzed by one or two populations analysis. The lifetimes are expressed as mean ± SEM for about 20 cells. For the two-population analysis, the long-lived lifetime was fixed to 2.37 ns, and the shorter lifetime as well as its amplitude were determined and expressed as mean ± SEM for about 20 cells. The lifetime of eGFP-Unr expressed alone was obtained through a one-population analysis. The bar scale represents 10 µm.

Since mCherry-Unr was seen to co-localize with NCp7-eGFP in fixed HeLa cells, we wondered if they could directly interact. To this end, we performed FRET-FLIM (Förster resonance energy transfer-Fluorescence Lifetime Imaging Microscopy) experiments on live cells co-expressing the two proteins of interest fused to eGFP and mCherry as fluorescence donor and acceptor, respectively. FRET between eGFP- and mCherry-labeled proteins only occurs when the two fluorophores are less than 8 nm apart, a distance corresponding to intermolecular protein–protein interactions [73,74,75]. FRET can be unambiguously quantified from the fluorescence lifetimes of eGFP-labeled proteins, measured at each pixel of the cell image, using the FLIM technique. Indeed, FRET resulted in a decrease in the eGFP lifetime that does not depend on the instrumentation or the concentration of the fluorophores, thus clearly demonstrating an interaction between the two proteins. As a control, cells expressing eGFP-Unr in the absence of NCp7 or Gag were imaged (Figure 6(A1)). The fluorescence lifetime (τ) of eGFP-Unr, as indicated through a color code in Figure 6A, was found to be very homogeneous over the cell, with a value of 2.37 ± 0.02 ns (Figure 6B), which is very similar to that of free eGFP (2.39 ± 0.08 ns; [49]). Co-expression of eGFP-Unr with NCp7 labeled by mCherry at its C-terminus (NCp7-mCherry) or N-terminus (mCherry-NCp7) resulted in a significant decrease in the fluorescence lifetime, as shown by the color change in respect to the control (Figure 6(A2,A3), respectively). This was even more visible when the eGFP-Unr lifetime distribution of all the measured pixels in 20 imaged cells was plotted (Figure 6B), showing a significantly shifted distribution towards a lower lifetime in the presence of mCherry-labeled NCp7. The average lifetime values in the presence of NCp7-mCherry or mCherry-NCp7 were 2.25 ± 0.05 ns and 2.21 ± 0.04 ns, respectively (Figure 6C), giving average FRET values of 5.1 and 6.8%, indicating an interaction between the two partners [73]. As the FRET percentages were nevertheless rather low, one possible explanation is that a significant amount of eGFP-Unr may not interact with NCp7 and exhibit thus a 2.4 ns lifetime that artificially increases the mean lifetime that is recovered using a mono-exponential model. To take into account the coexistence of bound and free eGFP-Unr populations, we analyzed the fluorescence decays with a two components model: F(t) = α_1_e^−t/τ1^ + α_2_e^−t/τ2^ where the long-lived lifetime τ_2_ was fixed at the lifetime of eGFP-Unr expressed alone (2.37 ns), while the short component τ_1_ and the populations (α_1_ and α_2_) associated with the two lifetimes were allowed to float. Using this bi-exponential fluorescence decay model, it appeared that 21–26% of the eGFP-Unr proteins were able to FRET and thus directly interacted with NCp7-mCherry and mCherry-NCp7, respectively, with a high FRET efficiency above 35%. This underlines that a significant fraction of Unr can interact with NCp7, bringing the two fluorophores close to each other.

### 3.6. Gag Interaction with Unr Visualized by FRET-FLIM

**Figure 7:**
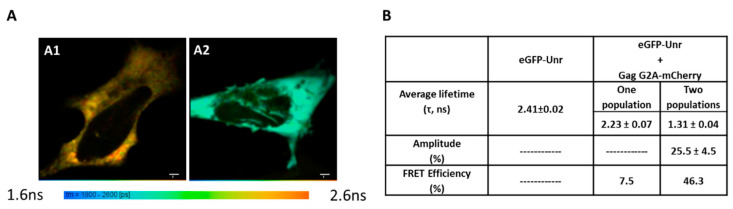
Unr/GagG2A interaction monitored by FRET-FLIM. HeLa cells were transfected with DNA constructs encoding eGFP-Unr in the absence (**A1**) and the presence of GagG2A-mCherry (**A2**). Live cells were imaged 24 h post-transfection. The eGFP fluorescence lifetimes were determined using a single-exponential model and converted into a color code ranging from blue (1.6 ns) to red (2.4 ns). (**B**) Fluorescence lifetimes of eGFP-Unr, in the presence of GagG2A-mCherry, were analyzed by one or two populations analysis. The lifetimes are expressed as mean ± SEM for about 20 cells. For the two populations analysis, the long lifetime was fixed to 2.41 ns, and the shorter lifetime as well as its amplitude were determined and expressed as mean ± SEM for about 20 cells. The bar scale represents 10 µm.

Because Gag is rapidly transported to the membrane shortly after its translation in the cytoplasm, we used to evidence the interaction with Unr, a previously published Gag-G2A (Glycine at position 2 mutated to Alanine) mutant lacking the N-terminus myristoylation motif responsible for the localization of Gag at the plasma membrane. This mutant shows a predominantly cytoplasmic localization associated with a low multimerization degree even though it retains its full RNA-binding ability. The fluorescence lifetime of eGFP-Unr was monitored in HeLa cells without (Figure 7(A1)) or with GagG2A-mCherry co-expression (Figure 7(A2)). Using a single population analysis, a significant decrease in the fluorescence lifetime of Unr-eGFP (from 2.41 ± 0.02 ns to 2.23 ± 0.07 ns) was observed (Figure 7B) when it was co-expressed with GagG2A-mCherry, corresponding to a FRET efficiency of 7.5%. As for NCp7, a two populations analysis revealed that around 25% of the total Unr-eGFP population was interacting with GagG2A with a strong FRET efficiency (46%).

### 3.7. NCp7 and Gag Interaction with Unr Monitored by Co-Immunoprecipitation

**Figure 8:**
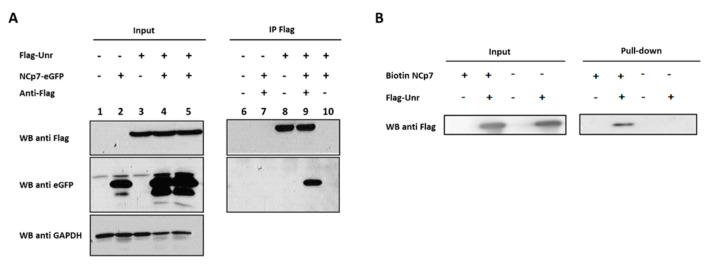
Co-immunoprecipitation of NCp7 and Unr. (**A**) Co-immunoprecipitation of NCp7-eGFP with Flag-Unr. 293T cell lysates expressing diverse combinations of proteins of interest after transient transfection (lanes 1–5) were subjected to immunoprecipitation with protein A beads linked to an anti-Flag antibody (lanes 6 to 9). The immunoprecipitates were analyzed by acrylamide gel SDS-PAGE and revealed by Western blot, using anti-eGFP and anti-Flag antibodies diluted to 1/10,000 and 1/4000, respectively. Homogenous loadings were checked using a Western blot against GAPDH (antibody diluted to 1/5000). Lane 10 corresponds to IP without anti-Flag antibody. (**B**) Pull-down of Flag-Unr with biotinylated NCp7 bound to streptavidin beads. 293T cells were transfected with an empty plasmid or a plasmid coding for Flag-Unr. Then, 48 h post transfection, cell lysates were added to streptavidin beads alone or streptavidin beads conjugated with biotinylated NC. Input (lanes 1–4) and the pull-down fractions (lanes 5–8) were analyzed by acrylamide gel SDS-PAGE and revealed by Western blot, using anti-Flag antibody (diluted 1/4000).

To confirm the interaction between Unr and Gag/NCp7, we performed co-immunoprecipitations (co-IP) on 293T cells co-transfected with constructs encoding Flag-Unr and NCp7-eGFP. The immunoprecipitates were analyzed by Western blot. All the fusion proteins were well overexpressed, as observed in the input lanes 1–5 in Figure 8A. As Flag-Unr is specifically immunoprecipitated by the anti-Flag antibody (in Figure 8A, compare lanes 8 and 9 with lane 10), the co-IP of NCp7-eGFP with Flag-Unr (compare lane 9 with lane 8) confirms that Flag-Unr and NCp7-eGFP interact specifically. The interaction between NCp7 and Unr was further confirmed by the co-IP of eGFP-Unr with Flag-NCp7 using an anti-eGFP antibody (data not shown). Moreover, we also performed a pull-down with a synthetic NCp7 biotinylated at its N-terminus (Biotin-NCp7) and fixed on streptavidin beads before the addition of 293T cell lysates overexpressing Flag-Unr protein (Figure 8B). A Western blot using an anti-Flag antibody confirmed the expression of Flag-Unr (Figure 8B lanes 2 and 4) and the specific presence of Unr in the pull-downed fraction with beads covered by Biotin-NCp7. Finally, no co-IP could be observed when a lysate of cells co-expressing Flag-Unr and NCp7-eGFP was treated with RNase just before performing the co-IP experiment (data not shown), suggesting that NCp7 interaction with Unr is RNA-dependent. Of note, although the co-IP data confirmed the interaction between Unr and NCp7, a more quantitative comparison with the FRET-FLIM data, especially regarding the interacting protein population, would be highly speculative as the cells were transfected with different constructs carrying different tags in the two datasets and the two techniques show different intrinsic limitations.

**Figure 9:**
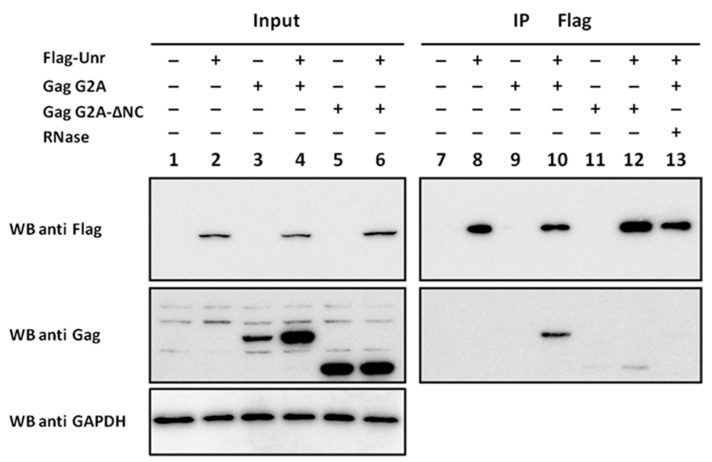
Co-immunoprecipitation of Gag G2A with Flag-Unr. 293T cell lysates expressing proteins of interest after transient transfection (lanes 1 to 6) were subjected to immunoprecipitation with anti-Flag antibody (diluted to 1/4000) conjugated to protein A beads. The immunoprecipitates were analyzed by acrylamide gel SDS-PAGE and revealed by Western blot, using anti-Gag antibody (diluted to 1/10,000) (lanes 7–13). In lane 13, the lysate was incubated for 30 min at room temperature with 100 μg/mL of RNAse A before addition of the antibody. Homogenous loadings were checked using a Western blot against GAPDH (diluted to 1/5000) (lanes 1–6).

As we measured an interaction by FRET/FLIM between GagG2A and Unr, we next validated this interaction by co-IP experiments. HeLa cells were transfected with different combinations of constructs coding for Flag-Unr, Gag G2A or a Gag G2A mutant with a deleted NC domain (Gag G2A-ΔNC). All the proteins were correctly expressed, as revealed by Western blot using an anti-GAPDH on input cell lysates (Figure 9, lanes 1–6). The lysates were subjected to immunoprecipitation with an anti-Flag antibody able to immunoprecipitate, specifically Flag-Unr (Figure 9, lanes 8, 10, 12 and 13). GagG2A specifically co-immunoprecipitated only when Flag-Unr was expressed (compare Figure 9 lane 10 with lane 9). In contrast, only a weak co-IP was observed when the NC domain of GagG2A was deleted (Figure 9 lane 12). Finally, no co-IP was seen when Flag-Unr was treated with RNase before the co-IP (Figure 9 lane 13), underlining a key role of RNA in the binding of Gag to Unr.

Altogether, these results confirmed the interaction of both NCp7 and Gag with Unr. They also indicated that Gag/Unr interaction is RNA-dependent and, to a large extent, mediated by the NC domain.

### 3.8. Unr Knockdown Reduces Infection by an HIV-1 Lentivector

**Figure 10:**
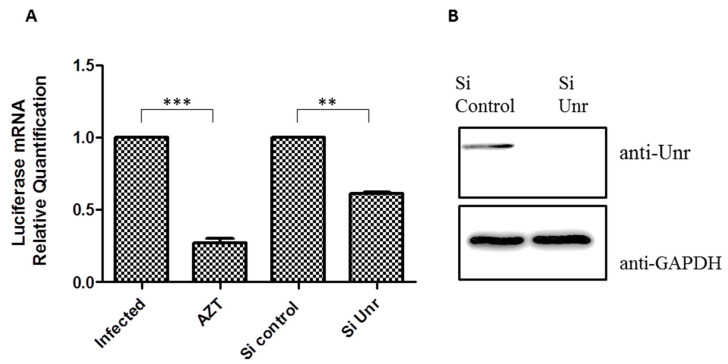
Inhibition of infection induced by Unr knockdown. (**A**) Firefly luciferase mRNA quantification by RT-qPCR after infection of HeLa cells with firefly-coding HIV-1 pseudoparticles. HeLa cells were either non-treated, treated with AZT, control siRNA or siRNA against Unr before infection. The graph shows the quantification of the luciferase mRNA relative to the mRNA of the housekeeping gene 18S. The quantification was realized using the delta Ct quantification method. Histograms represent the mean ± SEM of at least 3 independent experiments done in triplicate. ** *p* < 0.01; *** *p* < 0.001. (**B**) Control of Unr knockdown after siRNA treatment. Infections were done 48 h after siRNA transfection using either a control siRNA or a siRNA against Unr. Cell lysates were subjected to an SDS-PAGE and a Western blot against Unr (antibody diluted to 1/1000) or GAPDH (antibody diluted to 1/5000) as a loading control 48 h after infection. Of note, the relatively high mRNA level after AZT treatment is likely the consequence of the contribution of the genomic material of the incoming virus in the measurements.

To analyze the effect of Unr protein on HIV-1 viral infection, we used, as a model of the early stages of infection (post-entry to the integration), a non-replicative lentivirus pseudotyped with the VSV (Vesicular Stomatitis Virus) glycoprotein. In the lentivector genome, the viral genes were replaced by a Cytomegalovirus CMV promoter driving the synthesis of firefly luciferase mRNA. The level of cell infection could be monitored by measuring the enzymatic Fluc activity or the quantity of mRNA coding Fluc in infected cells. As Unr is part of the cellular translation machinery and the production of firefly luciferase protein could be influenced by Unr knockdown, we monitored cell infection by quantifying the Fluc mRNA by RT-qPCR and normalized it to that of the 18S housekeeping gene mRNA. HeLa cells were infected 48 h after transfection with either a control siRNA or siRNA against Unr. The reverse-transcriptase inhibitor AZT (zidovudine) was used as a positive control. In comparison to non-treated cells or cells treated with control siRNA, we observed a 30% decrease in infection after treatment with a siRNA against Unr. This decrease has to be compared with the 70% decrease observed when AZT was used (Figure 10A). The proper Unr knockdown was verified by Western blot in comparison to GAPDH (Figure 10B). Since this model only mimics the early phase of infection, the decrease in infection upon Unr knockdown suggests that Unr is important in the early phase of cell infection.

## 4. Discussion

Since Unr has been implicated in the IRES-mediated translation of Poliovirus and Rhinovirus in addition to cellular IRESs [33,34,35,36], we hypothesized in this paper that Unr could also be implicated in the HIV-1 IRES-dependent translation. In fact, the HIV-1 5′-UTR bears two IRESs, one in the 5′-UTR found in the gRNA and all viral mRNAs and a second one in the Gag coding region which drives the synthesis of Gagp55 and a 40 kDa truncated form of Gag. Since the HIV-1 IRES was shown to be not functional in RRL unless supplemented with cellular extracts, especially from G2/M blocked cells, it was assumed to be regulated by cellular factors. Among the factors reported to increase HIV-1 IRES activity are the heterogeneous nuclear ribonucleoprotein A1 (hnRNPA1), eIF5A, S25, Staufen [16,76,77,78] and the Rev co-factors DDX3 and hRIP [16,17]. However, the regulatory mechanism is likely to differ among those factors.

In this study, we used a dual luciferase system based on cell transfection with a bicistronic construct in which the firefly luciferase and the renilla luciferase expressions are under the control of the cap-dependent and the HIV-1 IRES translation, respectively. We showed for the first time that Unr is an ITAF having a stimulatory action on the activity of the HIV-1 IRES. Moreover, we identified a single-stranded region between nucleotides 205–215 of the 5′-UTR as a binding site of HIV-1 IRES. This region is located at the bottom of the PBS loop and close to the NC binding sites [68,79]. Mutations in this sequence greatly affected the IRES activity. This decrease is probably not linked to a loss of the proper folding of the HIV-1 IRES, as this IRES was shown to be highly resistant to mutations and deletions [14,15,18,66]. Three out of four point mutations (mut 211, 213 and 214) presented a decrease in the IRES activity and response to Unr, with mut211 being almost totally inactive and non-responsive to Unr overexpression. On the other hand, mut212 was more active than the WT and showed a more sensitive response to Unr overexpression. The functional implication of this sequence is in line with a previous study [15], which identified sequence 202–217 as being able to increase the IRES activity. Furthermore, the SHAPE reactivity of nucleotides 211–213 was shown to be modified upon aldrithiol-2 (AT-2) treatment, compromising the NC-RNA interaction [15]. Therefore, we speculate that this RNA region important for Unr-stimulated IRES activity also binds NCp7, which may explain that NCp7, in our dual-luciferase assay, does not present any stimulatory effect and even counteracts the Unr action.

Interestingly, Gag was observed to behave differently to NCp7, showing a stimulatory effect on HIV-1 IRES, which is additive to that of Unr. This effect of Gag is in line with the ability of this protein to stimulate its own translation when expressed at low concentration [80], although it has been attributed to the matrix domain rather than the NC domain. The IRES stimulation observed with Gag in contrast to NCp7, as well as the stimulatory effect of Gag on its own translation, could be tentatively explained by the differences in the nucleic acid chaperone properties of the two proteins, especially at low protein concentrations [69,81,82]. Using FRET-FLIM and co-immunoprecipitation experiments, we clearly evidenced the interaction of both mature NCp7 and Gag with Unr, confirming the physical interaction between these proteins predicted by Jäger et al. [54]. We further showed that this interaction is RNA dependent and that, in the case of Gag, it is mainly mediated by its NC domain. The RNA dependence of the interaction is consistent with the fact that Unr [65,83] as well as NCp7 and Gag [84,85] are RNA-binding proteins and that other NC interactions with host proteins were reported to be RNA dependent [86]. This RNA dependence raises the possibility that the observed interaction between the proteins may simply correspond to their random binding to the same RNAs. This hypothesis could be easily refuted by our FRET-FLIM data showing that up to 26% of the eGFP-Unr proteins were able to FRET with mCherry-labelled NCp7 with a FRET efficiency greater than 35%. Assuming a Förster distance of 5 nm for the pair (eGFP, mCherry), this means that the average distance between the two fluorescent proteins is less than 5.5 nm. Knowing that the two fluorescent proteins have a similar molecular weight and that eGFP resembles a cylinder with a length of 4.2 nm and a diameter of 2.4 nm (Hink et al., 2000), our data demonstrate that the two fluorescent proteins should be in physical contact or separated by less than 2 nm. This can obviously only be achieved by a direct interaction between the two chimeric proteins and not by their random binding to nucleic acids. The second evidence comes from our data using synthetic biotinylated NCp7 (Figure 8B), showing that Unr can be specifically pulled down from a lysate of cells expressing Flag-Unr by this bead-coupled synthetic NCp7. Again, such an efficient pull down by random binding of NCp7 and Unr to the same RNAs would be highly unlikely. Therefore, RNA can be seen as a scaffold that promotes the direct interaction between Gag or NC with UNR. The functional implication of Unr in HIV-1 infection was tested using non-replicative lentiviral VSV- pseudotyped particles which mimic the early phase of the infection (entry to integration). Unr knockdown induced a 30% decrease in infection, suggesting that Unr is implicated in the early phase of the viral life cycle, most probably in the reverse transcription or integration step. It can be speculated that due to its ITAF properties, Unr acts as an RNA chaperone so that via its interaction with NCp7, both proteins can chaperone the reverse transcription.

Early in the late phase of infection, HIV-1 proteins are translated by a cap-dependent mechanism, which is then shut down by a cell cycle G2/M arrest [87]. At this point, Unr, being overexpressed in G2/M, may stimulate the HIV-1 IRES in synergy with Gag. Thus, Gag is thought to recruit Unr to the viral RNA specifically encapsidated by Gag [88] in order to optimize its translation and sustain an important viral protein production. In contrast, as the viral RNA is thought to be entirely coated by NCp7 in the reverse transcription complex and as NCp7 counteracts the Unr stimulating activity on the IRES, it may be hypothesized that NCp7 protects the gRNA from Unr-mediated IRES stimulation.

## 5. Conclusions

In conclusion, we validated by co-immunoprecipitation and FRET-FLIM the interaction of Unr with NCp7 as a mature protein or as a domain of Gag. The interaction was found to be RNA-dependent. We also demonstrated the importance of Unr in the early phases of the viral infection and showed for the first time the role of Unr as an ITAF for HIV-1 IRES. The effect of Unr on IRES activity was found to be counteracted by NCp7 and additive to Gag.

## Figures and Tables

**Table 1:** List of primers used to generate IRES mutants.

IRES mut rev	(Phos)TGTTCGGGCGCCACTGCTAGAG
IRES mut U fwd	(Phos)GGGACACTTGAAAGCGATTCATAAGCCAGAGGAG
IRES mut C fwd	(Phos)GGGACTTGAAAGCGACCAGCAAGCCAGAGGAG
IRES mut 211 fwd	(Phos)GGGACTTGAAAGCGAAGGTAAAGCCAGAGGAG
IRES mut 212 fwd	(Phos)GGGACTTGAAAGCGAAACTAAAGCCAGAGGAG
IRES mut 213 fwd	(Phos)GGGACTTGAAAGCGAAAGAAAAGCCAGAGGAG
IRES mut 214 fwd	(Phos)GGGACTTGAAAGCGAAAGTGAAGCCAGAGGAG

**Table 2:** Mutations in the HIV-1 IRES sequence to identify Unr binding site.

Clone	Sequence
Wild-type sequence	210 AAGUA 214
mutU	210 **UUCAU** 214
mutC	210 **CCAGC** 214
mut211	210 A**G**GUA 214
mut212	210 AA**C**UA 214
mut213	210 AAG**A**A 214
mut214	210 AAGU**G** 214

## Data Availability

Not applicable.

## References

[1] Keane S.C., Heng X., Lu K., Kharytonchyk S., Ramakrishnan V., Carter G., Barton S., Hosic A., Florwick A., Santos J. (2015). RNA Structure. Structure of the HIV-1 RNA Packaging Signal. Science.

[2] Brown J.D., Kharytonchyk S., Chaudry I., Iyer A.S., Carter H., Becker G., Desai Y., Glang L., Choi S.H., Singh K. (2020). Structural Basis for Transcriptional Start Site Control of HIV-1 RNA Fate. Science.

[3] Ding P., Kharytonchyk S., Waller A., Mbaekwe U., Basappa S., Kuo N., Frank H.M., Quasney C., Kidane A., Swanson C. (2020). Identification of the Initial Nucleocapsid Recognition Element in the HIV-1 RNA Packaging Signal. Proc. Natl. Acad. Sci. USA.

[4] Miele G., Mouland A., Harrison G.P., Cohen E., Lever A.M. (1996). The Human Immunodeficiency Virus Type 1 5′ Packaging Signal Structure Affects Translation but Does Not Function as an Internal Ribosome Entry Site Structure. J. Virol..

[5] Berkhout B., Arts K., Abbink T.E.M. (2011). Ribosomal Scanning on the 5′-Untranslated Region of the Human Immunodeficiency Virus RNA Genome. Nucleic Acids Res..

[6] Ricci E.P., Soto Rifo R., Herbreteau C.H., Decimo D., Ohlmann T. (2008). Lentiviral RNAs Can Use Different Mechanisms for Translation Initiation. Biochem. Soc. Trans..

[7] de Breyne S., Ohlmann T. (2019). Focus on Translation Initiation of the HIV-1 MRNAs. Int. J. Mol. Sci..

[8] Buck C.B., Shen X., Egan M.A., Pierson T.C., Walker C.M., Siliciano R.F. (2001). The Human Immunodeficiency Virus Type 1 Gag Gene Encodes an Internal Ribosome Entry Site. J. Virol..

[9] Herbreteau C.H., Weill L., Décimo D., Prévôt D., Darlix J.-L., Sargueil B., Ohlmann T. (2005). HIV-2 Genomic RNA Contains a Novel Type of IRES Located Downstream of Its Initiation Codon. Nat. Struct. Mol. Biol..

[10] Locker N., Chamond N., Sargueil B. (2011). A Conserved Structure within the HIV Gag Open Reading Frame That Controls Translation Initiation Directly Recruits the 40S Subunit and EIF3. Nucleic Acids Res..

[11] Weill L., James L., Ulryck N., Chamond N., Herbreteau C.H., Ohlmann T., Sargueil B. (2010). A New Type of IRES within Gag Coding Region Recruits Three Initiation Complexes on HIV-2 Genomic RNA. Nucleic Acids Res..

[12] Brasey A., Lopez-Lastra M., Ohlmann T., Beerens N., Berkhout B., Darlix J.-L., Sonenberg N. (2003). The Leader of Human Immunodeficiency Virus Type 1 Genomic RNA Harbors an Internal Ribosome Entry Segment That Is Active during the G2/M Phase of the Cell Cycle. J. Virol..

[13] Rivas-Aravena A., Ramdohr P., Vallejos M., Valiente-Echeverría F., Dormoy-Raclet V., Rodríguez F., Pino K., Holzmann C., Huidobro-Toro J.P., Gallouzi I.-E. (2009). The Elav-like Protein HuR Exerts Translational Control of Viral Internal Ribosome Entry Sites. Virology.

[14] Vallejos M., Deforges J., Plank T.-D.M., Letelier A., Ramdohr P., Abraham C.G., Valiente-Echeverría F., Kieft J.S., Sargueil B., López-Lastra M. (2011). Activity of the Human Immunodeficiency Virus Type 1 Cell Cycle-Dependent Internal Ribosomal Entry Site Is Modulated by IRES Trans-Acting Factors. Nucleic Acids Res..

[15] Gendron K., Ferbeyre G., Heveker N., Brakier-Gingras L. (2011). The Activity of the HIV-1 IRES Is Stimulated by Oxidative Stress and Controlled by a Negative Regulatory Element. Nucleic Acids Res..

[16] Liu J., Henao-Mejia J., Liu H., Zhao Y., He J.J. (2011). Translational Regulation of HIV-1 Replication by HIV-1 Rev Cellular Cofactors Sam68, EIF5A, HRIP, and DDX3. J. Neuroimmune Pharmacol..

[17] Monette A., Ajamian L., López-Lastra M., Mouland A.J. (2009). Human Immunodeficiency Virus Type 1 (HIV-1) Induces the Cytoplasmic Retention of Heterogeneous Nuclear Ribonucleoprotein A1 by Disrupting Nuclear Import: Implications for HIV-1 Gene Expression. J. Biol. Chem..

[18] Vallejos M., Carvajal F., Pino K., Navarrete C., Ferres M., Huidobro-Toro J.P., Sargueil B., López-Lastra M. (2012). Functional and Structural Analysis of the Internal Ribosome Entry Site Present in the MRNA of Natural Variants of the HIV-1. PLoS ONE.

[19] Borman A., Howell M.T., Patton J.G., Jackson R.J. (1993). The Involvement of a Spliceosome Component in Internal Initiation of Human Rhinovirus RNA Translation. J. Gen. Virol..

[20] Meerovitch K., Svitkin Y.V., Lee H.S., Lejbkowicz F., Kenan D.J., Chan E.K., Agol V.I., Keene J.D., Sonenberg N. (1993). La Autoantigen Enhances and Corrects Aberrant Translation of Poliovirus RNA in Reticulocyte Lysate. J. Virol..

[21] Mitchell S.A., Brown E.C., Coldwell M.J., Jackson R.J., Willis A.E. (2001). Protein Factor Requirements of the Apaf-1 Internal Ribosome Entry Segment: Roles of Polypyrimidine Tract Binding Protein and Upstream of N-Ras. Mol. Cell Biol..

[22] Monette A., Valiente-Echeverría F., Rivero M., Cohen É.A., Lopez-Lastra M., Mouland A.J. (2013). Dual Mechanisms of Translation Initiation of the Full-Length HIV-1 MRNA Contribute to Gag Synthesis. PLoS ONE.

[23] Amorim R., Costa S.M., Cavaleiro N.P., da Silva E.E., da Costa L.J. (2014). HIV-1 Transcripts Use IRES-Initiation under Conditions Where Cap-Dependent Translation Is Restricted by Poliovirus 2A Protease. PLoS ONE.

[24] Rogel M.E., Wu L.I., Emerman M. (1995). The Human Immunodeficiency Virus Type 1 Vpr Gene Prevents Cell Proliferation during Chronic Infection. J. Virol..

[25] King H.A., Cobbold L.C., Willis A.E. (2010). The Role of IRES Trans-Acting Factors in Regulating Translation Initiation. Biochem. Soc. Trans..

[26] Balvay L., Soto Rifo R., Ricci E.P., Decimo D., Ohlmann T. (2009). Structural and Functional Diversity of Viral IRESes. Biochim. Biophys. Acta.

[27] Filbin M.E., Kieft J.S. (2009). Toward a Structural Understanding of IRES RNA Function. Curr. Opin. Struct. Biol..

[28] Fitzgerald K.D., Semler B.L. (2009). Bridging IRES Elements in MRNAs to the Eukaryotic Translation Apparatus. Biochim. Biophys. Acta.

[29] Jacquemin-Sablon H., Triqueneaux G., Deschamps S., le Maire M., Doniger J., Dautry F. (1994). Nucleic Acid Binding and Intracellular Localization of Unr, a Protein with Five Cold Shock Domains. Nucleic Acids Res..

[30] Ferrer N., Garcia-Espana A., Jeffers M., Pellicer A. (1999). The Unr Gene: Evolutionary Considerations and Nucleic Acid-Binding Properties of Its Long Isoform Product. DNA Cell Biol..

[31] Saltel F., Giese A., Azzi L., Elatmani H., Costet P., Ezzoukhry Z., Dugot-Senant N., Miquerol L., Boussadia O., Wodrich H. (2017). Unr Defines a Novel Class of Nucleoplasmic Reticulum Involved in MRNA Translation. J. Cell Sci..

[32] Hollmann N.M., Jagtap P.K.A., Masiewicz P., Guitart T., Simon B., Provaznik J., Stein F., Haberkant P., Sweetapple L.J., Villacorta L. (2020). Pseudo-RNA-Binding Domains Mediate RNA Structure Specificity in Upstream of N-Ras. Cell Rep..

[33] Boussadia O., Niepmann M., Créancier L., Prats A.-C., Dautry F., Jacquemin-Sablon H. (2003). Unr Is Required in Vivo for Efficient Initiation of Translation from the Internal Ribosome Entry Sites of Both Rhinovirus and Poliovirus. J. Virol..

[34] Brown E.C., Jackson R.J. (2004). All Five Cold-Shock Domains of Unr (Upstream of N-Ras) Are Required for Stimulation of Human Rhinovirus RNA Translation. J. Gen. Virol..

[35] Mitchell S.A., Spriggs K.A., Coldwell M.J., Jackson R.J., Willis A.E. (2003). The Apaf-1 Internal Ribosome Entry Segment Attains the Correct Structural Conformation for Function via Interactions with PTB and Unr. Mol. Cell.

[36] Tinton S.A., Schepens B., Bruynooghe Y., Beyaert R., Cornelis S. (2005). Regulation of the Cell-Cycle-Dependent Internal Ribosome Entry Site of the PITSLRE Protein Kinase: Roles of Unr (Upstream of N-Ras) Protein and Phosphorylated Translation Initiation Factor EIF-2alpha. Biochem. J..

[37] Clever J., Sassetti C., Parslow T.G. (1995). RNA Secondary Structure and Binding Sites for Gag Gene Products in the 5′ Packaging Signal of Human Immunodeficiency Virus Type 1. J. Virol..

[38] Clever J.L., Miranda D., Parslow T.G. (2002). RNA Structure and Packaging Signals in the 5′ Leader Region of the Human Immunodeficiency Virus Type 1 Genome. J. Virol..

[39] Dannull J., Surovoy A., Jung G., Moelling K. (1994). Specific Binding of HIV-1 Nucleocapsid Protein to PSI RNA in Vitro Requires N-Terminal Zinc Finger and Flanking Basic Amino Acid Residues. EMBO J..

[40] Darlix J.-L., Garrido J.L., Morellet N., Mély Y., de Rocquigny H. (2007). Properties, Functions, and Drug Targeting of the Multifunctional Nucleocapsid Protein of the Human Immunodeficiency Virus. Adv. Pharmacol..

[41] Darlix J.L., Lapadat-Tapolsky M., de Rocquigny H., Roques B.P. (1995). First Glimpses at Structure-Function Relationships of the Nucleocapsid Protein of Retroviruses. J. Mol. Biol..

[42] Thomas J.A., Gorelick R.J. (2008). Nucleocapsid Protein Function in Early Infection Processes. Virus Res..

[43] Darlix J.-L., Godet J., Ivanyi-Nagy R., Fossé P., Mauffret O., Mély Y. (2011). Flexible Nature and Specific Functions of the HIV-1 Nucleocapsid Protein. J. Mol. Biol..

[44] Berkowitz R., Fisher J., Goff S.P. (1996). RNA Packaging. Curr. Top. Microbiol. Immunol..

[45] Sakuragi J., Shioda T., Panganiban A.T. (2001). Duplication of the Primary Encapsidation and Dimer Linkage Region of Human Immunodeficiency Virus Type 1 RNA Results in the Appearance of Monomeric RNA in Virions. J. Virol..

[46] Ding P., Kharytonchyk S., Kuo N., Cannistraci E., Flores H., Chaudhary R., Sarkar M., Dong X., Telesnitsky A., Summers M.F. (2021). 5′-Cap Sequestration Is an Essential Determinant of HIV-1 Genome Packaging. Proc. Natl. Acad. Sci. USA.

[47] Berkhout B., van Wamel J.L. (1996). Role of the DIS Hairpin in Replication of Human Immunodeficiency Virus Type 1. J. Virol..

[48] Clever J.L., Parslow T.G. (1997). Mutant Human Immunodeficiency Virus Type 1 Genomes with Defects in RNA Dimerization or Encapsidation. J. Virol..

[49] El Meshri S.E., Dujardin D., Godet J., Richert L., Boudier C., Darlix J.L., Didier P., Mély Y., de Rocquigny H. (2015). Role of the Nucleocapsid Domain in HIV-1 Gag Oligomerization and Trafficking to the Plasma Membrane: A Fluorescence Lifetime Imaging Microscopy Investigation. J. Mol. Biol..

[50] de Rocquigny H., El Meshri S.E., Richert L., Didier P., Darlix J.-L., Mély Y. (2014). Role of the Nucleocapsid Region in HIV-1 Gag Assembly as Investigated by Quantitative Fluorescence-Based Microscopy. Virus Res..

[51] Cimarelli A., Sandin S., Höglund S., Luban J. (2000). Basic Residues in Human Immunodeficiency Virus Type 1 Nucleocapsid Promote Virion Assembly via Interaction with RNA. J. Virol..

[52] Dorfman T., Luban J., Goff S.P., Haseltine W.A., Göttlinger H.G. (1993). Mapping of Functionally Important Residues of a Cysteine-Histidine Box in the Human Immunodeficiency Virus Type 1 Nucleocapsid Protein. J. Virol..

[53] Gorelick R.J., Nigida S.M., Bess J.W., Arthur L.O., Henderson L.E., Rein A. (1990). Noninfectious Human Immunodeficiency Virus Type 1 Mutants Deficient in Genomic RNA. J. Virol..

[54] Jäger S., Cimermancic P., Gulbahce N., Johnson J.R., McGovern K.E., Clarke S.C., Shales M., Mercenne G., Pache L., Li K. (2011). Global Landscape of HIV-Human Protein Complexes. Nature.

[55] Boutant E., Bonzi J., Anton H., Nasim M.B., Cathagne R., Réal E., Dujardin D., Carl P., Didier P., Paillart J.-C. (2020). Zinc Fingers in HIV-1 Gag Precursor Are Not Equivalent for GRNA Recruitment at the Plasma Membrane. Biophys. J..

[56] De Rocquigny H., Gabus C., Vincent A., Fournié-Zaluski M.C., Roques B., Darlix J.L. (1992). Viral RNA Annealing Activities of Human Immunodeficiency Virus Type 1 Nucleocapsid Protein Require Only Peptide Domains Outside the Zinc Fingers. Proc. Natl. Acad. Sci. USA.

[57] Simon J.H., Fouchier R.A., Southerling T.E., Guerra C.B., Grant C.K., Malim M.H. (1997). The Vif and Gag Proteins of Human Immunodeficiency Virus Type 1 Colocalize in Infected Human T Cells. J. Virol..

[58] Fouchier R.A., Meyer B.E., Simon J.H., Fischer U., Malim M.H. (1997). HIV-1 Infection of Non-Dividing Cells: Evidence That the Amino-Terminal Basic Region of the Viral Matrix Protein Is Important for Gag Processing but Not for Post-Entry Nuclear Import. EMBO J..

[59] Azoulay J., Clamme J.P., Darlix J.L., Roques B.P., Mély Y. (2003). Destabilization of the HIV-1 Complementary Sequence of TAR by the Nucleocapsid Protein through Activation of Conformational Fluctuations. J. Mol. Biol..

[60] Clamme J.-P., Krishnamoorthy G., Mély Y. (2003). Intracellular Dynamics of the Gene Delivery Vehicle Polyethylenimine during Transfection: Investigation by Two-Photon Fluorescence Correlation Spectroscopy. Biochim. Biophys. Acta.

[61] Livak K.J., Schmittgen T.D. (2001). Analysis of Relative Gene Expression Data Using Real-Time Quantitative PCR and the 2(-Delta Delta C(T)) Method. Methods.

[62] Valiente-Echeverría F., Vallejos M., Monette A., Pino K., Letelier A., Huidobro-Toro J.P., Mouland A.J., López-Lastra M. (2013). A Cis-Acting Element Present within the Gag Open Reading Frame Negatively Impacts on the Activity of the HIV-1 IRES. PLoS ONE.

[63] Berkhout B. (1996). Structure and Function of the Human Immunodeficiency Virus Leader RNA. Prog. Nucleic Acid. Res. Mol. Biol..

[64] Coldwell M.J., Mitchell S.A., Stoneley M., MacFarlane M., Willis A.E. (2000). Initiation of Apaf-1 Translation by Internal Ribosome Entry. Oncogene.

[65] Triqueneaux G., Velten M., Franzon P., Dautry F., Jacquemin-Sablon H. (1999). RNA Binding Specificity of Unr, a Protein with Five Cold Shock Domains. Nucleic Acids Res..

[66] Plank T.-D.M., Whitehurst J.T., Kieft J.S. (2013). Cell Type Specificity and Structural Determinants of IRES Activity from the 5′ Leaders of Different HIV-1 Transcripts. Nucleic Acids Res..

[67] Abd El-Wahab E.W., Smyth R.P., Mailler E., Bernacchi S., Vivet-Boudou V., Hijnen M., Jossinet F., Mak J., Paillart J.-C., Marquet R. (2014). Specific Recognition of the HIV-1 Genomic RNA by the Gag Precursor. Nat. Commun..

[68] Wilkinson K.A., Gorelick R.J., Vasa S.M., Guex N., Rein A., Mathews D.H., Giddings M.C., Weeks K.M. (2008). High-Throughput SHAPE Analysis Reveals Structures in HIV-1 Genomic RNA Strongly Conserved across Distinct Biological States. PLoS Biol..

[69] Wu T., Datta S.A.K., Mitra M., Gorelick R.J., Rein A., Levin J.G. (2010). Fundamental Differences between the Nucleic Acid Chaperone Activities of HIV-1 Nucleocapsid Protein and Gag or Gag-Derived Proteins: Biological Implications. Virology.

[70] Cen S., Khorchid A., Gabor J., Rong L., Wainberg M.A., Kleiman L. (2000). Roles of Pr55(Gag) and NCp7 in TRNA(3)(Lys) Genomic Placement and the Initiation Step of Reverse Transcription in Human Immunodeficiency Virus Type 1. J. Virol..

[71] Cruceanu M., Urbaneja M.A., Hixson C.V., Johnson D.G., Datta S.A., Fivash M.J., Stephen A.G., Fisher R.J., Gorelick R.J., Casas-Finet J.R. (2006). Nucleic Acid Binding and Chaperone Properties of HIV-1 Gag and Nucleocapsid Proteins. Nucleic Acids Res..

[72] Karnib H., Nadeem M.F., Humbert N., Sharma K.K., Grytsyk N., Tisné C., Boutant E., Lequeu T., Réal E., Boudier C. (2020). The Nucleic Acid Chaperone Activity of the HIV-1 Gag Polyprotein Is Boosted by Its Cellular Partner RPL7: A Kinetic Study. Nucleic Acids Res..

[73] Voss T.C., Demarco I.A., Day R.N. (2005). Quantitative Imaging of Protein Interactions in the Cell Nucleus. Biotechniques.

[74] Day R.N., Periasamy A., Schaufele F. (2001). Fluorescence Resonance Energy Transfer Microscopy of Localized Protein Interactions in the Living Cell Nucleus. Methods.

[75] Bastiaens P.I., Squire A. (1999). Fluorescence Lifetime Imaging Microscopy: Spatial Resolution of Biochemical Processes in the Cell. Trends Cell Biol..

[76] Barrera A., Ramos H., Vera-Otarola J., Fernández-García L., Angulo J., Olguín V., Pino K., Mouland A.J., López-Lastra M. (2020). Post-Translational Modifications of HnRNP A1 Differentially Modulate Retroviral IRES-Mediated Translation Initiation. Nucleic Acids Res..

[77] Ramos H., Monette A., Niu M., Barrera A., López-Ulloa B., Fuentes Y., Guizar P., Pino K., DesGroseillers L., Mouland A.J. (2022). The Double-Stranded RNA-Binding Protein, Staufen1, Is an IRES-Transacting Factor Regulating HIV-1 Cap-Independent Translation Initiation. Nucleic Acids Res..

[78] Carvajal F., Vallejos M., Walters B., Contreras N., Hertz M.I., Olivares E., Cáceres C.J., Pino K., Letelier A., Thompson S.R. (2016). Structural Domains within the HIV-1 MRNA and the Ribosomal Protein S25 Influence Cap-Independent Translation Initiation. FEBS J..

[79] Levin J.G., Mitra M., Mascarenhas A., Musier-Forsyth K. (2010). Role of HIV-1 Nucleocapsid Protein in HIV-1 Reverse Transcription. RNA Biol..

[80] Anderson E.C., Lever A.M.L. (2006). Human Immunodeficiency Virus Type 1 Gag Polyprotein Modulates Its Own Translation. J. Virol..

[81] Roldan A., Warren O.U., Russell R.S., Liang C., Wainberg M.A. (2005). A HIV-1 Minimal Gag Protein Is Superior to Nucleocapsid at in Vitro Annealing and Exhibits Multimerization-Induced Inhibition of Reverse Transcription. J. Biol. Chem..

[82] Jones C.P., Datta S.A.K., Rein A., Rouzina I., Musier-Forsyth K. (2011). Matrix Domain Modulates HIV-1 Gag’s Nucleic Acid Chaperone Activity via Inositol Phosphate Binding. J. Virol..

[83] Hunt S.L., Hsuan J.J., Totty N., Jackson R.J. (1999). Unr, a Cellular Cytoplasmic RNA-Binding Protein with Five Cold-Shock Domains, Is Required for Internal Initiation of Translation of Human Rhinovirus RNA. Genes Dev..

[84] Athavale S.S., Ouyang W., McPike M.P., Hudson B.S., Borer P.N. (2010). Effects of the Nature and Concentration of Salt on the Interaction of the HIV-1 Nucleocapsid Protein with SL3 RNA. Biochemistry.

[85] Fisher R.J., Rein A., Fivash M., Urbaneja M.A., Casas-Finet J.R., Medaglia M., Henderson L.E. (1998). Sequence-Specific Binding of Human Immunodeficiency Virus Type 1 Nucleocapsid Protein to Short Oligonucleotides. J. Virol..

[86] Burnett A., Spearman P. (2007). APOBEC3G Multimers Are Recruited to the Plasma Membrane for Packaging into Human Immunodeficiency Virus Type 1 Virus-like Particles in an RNA-Dependent Process Requiring the NC Basic Linker. J. Virol..

[87] Zhao R.Y., Li G., Bukrinsky M.I. (2011). Vpr-Host Interactions during HIV-1 Viral Life Cycle. J. Neuroimmune Pharmacol..

[88] Webb J.A., Jones C.P., Parent L.J., Rouzina I., Musier-Forsyth K. (2013). Distinct Binding Interactions of HIV-1 Gag to Psi and Non-Psi RNAs: Implications for Viral Genomic RNA Packaging. RNA.

